# Genomics and transcriptomics reveal new molecular mechanism of vibriosis resistance in fish

**DOI:** 10.3389/fimmu.2022.974604

**Published:** 2022-09-29

**Authors:** Qian Zhou, Yadong Chen, Zhangfan Chen, Lei Wang, Xinran Ma, Jie Wang, Qihao Zhang, Songlin Chen

**Affiliations:** ^1^ Yellow Sea Fisheries Research Institute, Chinese Academy of Fishery Sciences; Key Laboratory for Sustainable Development of Marine Fisheries, Ministry of Agriculture, Qingdao, China; ^2^ Laboratory for Marine Fisheries Science and Food Production Processes, Pilot National Laboratory for Marine Science and Technology, Qingdao, China; ^3^ Shandong Key Laboratory for Marine Fishery Biotechnology and Genetic Breeding, Qingdao, China; ^4^ College of Life Science, Qingdao University, Qingdao, China

**Keywords:** vibriosis resistance, molecular mechanism, selective sweep, RNA-Seq, fish disease control, *Cynoglossus semilaevis*

## Abstract

Infectious diseases have caused dramatic production decline and economic loss for fish aquaculture. However, the poor understanding of fish disease resistance severely hampered disease prevention. Chinese tongue sole (*Cynoglossus semilaevis*) is an important economic flatfish suffering from vibriosis. Here we used genomic, transcriptomic and experimental approaches to investigate the molecular genetic mechanisms underlying fish vibriosis resistance. A genome-wide comparison revealed that the genes under selective sweeps were enriched for glycosaminoglycan (GAG) chondroitin sulfate (CS)/dermatan sulfate (DS) metabolism. Transcriptomic analyses prioritized synergic gene expression patterns in this pathway, which may lead to an increased CS/DS content in the resistant family. Further experimental evidence showed that carbohydrate sulfotransferases 12 (Chst12), a key enzyme for CS/DS biosynthesis, has a direct antibacterial activity. To the best of our knowledge, this is the first report that the *chst12* gene has a bactericidal effect. In addition, CS/DS is a major component of the extracellular matrix (ECM) and the selection signatures and fine-tuned gene expressions of ECM-receptor interaction genes indicated a modification in the ECM structure with an enhancement of the barrier function. Furthermore, functional studies conducted on Col6a2, encoding a collagen gene which constitutes the ECM, pointed to that it may act as a cellular receptor for *Vibrio* pathogens, thus plays an important role for the *Vibrio* invasion. Taken together, these findings provide new insights into the molecular protective mechanism underlying vibriosis resistance in fish, which offers crucial genomic resources for the resistant germplasm breeding and infectious disease control in fish culturing.

## Introduction

Currently, the global food production and security is facing great challenges. Aquaculture plays an increasingly important role in nutrition and food supply. However, infectious diseases are recognized as a major cause of mortality and constitute a major global threat for the production of fish farming (1), and the success and sustainability of fish aquaculture largely depends on the control of diseases (2). Genetic breeding of fish with improved diseases resistance remains a highly sought-after objective in aquaculture (3), providing effective and long-term control over disease problem. To achieve the selective breeding and disease control, it is important to understand the molecular mechanisms determining the resistance of fish to pathogenic microbes.

Conceptually, “disease resistance” refers to the host’s ability to reduce pathogen invasion (limitation of pathogen entry into the targe tissue and replication) (1), which in fish encompasses a variety of mechanisms including maintenance of epithelial barriers and the mucus coat; nonspecific cellular factors such as phagocytosis by macrophages and neutrophils; nonspecific humoral factors such as lysozyme, complement, and transferrin; and specific humoral and cellular immunity (4). A number of studies have documented the genetic variations and genes associated with disease resistance in fish. Quantitative trait locus (QTL) mappings and genome-wide association studies (GWAS) allowed detection of the single-nucleotide polymorphisms (SNPs) and genes associated with disease resistance in many fish, such as Atlantic salmon, rainbow trout (2, 5) and Chinese tongue sole (6). Comparative transcriptome analyses of resistant and susceptible fish upon pathogenic infections indicated that transcriptional responses induced by various pathogens generally involved essentially the same genes and pathways in immune systems, such as complement, immune signaling transduction pathways and a number of enzymes and chemokines among Atlantic salmon (7, 8), rainbow trout (9), common carp (10) and Chinese tongue sole (11). While these studies have shown that the resistant and susceptible fish have different genetic architecture and distinct molecular responses after temporary infections, a crucial question that how the fish disease resistance emerges and why the resistant fish can resist the pathogenic infections remains poorly resolved.

Vibriosis, caused by the *Vibrio* genera species such as *V. anguillarum*, *V. alginolyticus*, *V. harveyi*, and *V. splendidus*, is one of the most detrimental infectious diseases for various marine fish and invertebrate. Outbreaks of vibriosis result to 50-100% mortalities in different fish. Chinese tongues sole (*Cynoglossus semilaevis*) is an important and widely cultivated economic flatfish species with delicious taste and superior nutritive value, which is recorded as one of the nine varieties in the national marine fish industry technology system of China (https://www.cafs.ac.cn/info/1024/38584.htm). *C. semilaevis* has suffered from striking production decline caused by its dominant bacterial pathogen *V. harveyi*. In our previous work, we have conducted a successive selective breeding for more than 10 years and produced robust *C. semilaevis* families with high vibriosis resistance (12). This constant selection practice provides a unique opportunity for tracing the evolutionary and molecular basis underlying the acquisition of vibriosis resistance in fish, using the pre-selection and post-selection individuals. It is proposed that divergence in both gene sequence frequencies and gene expressions underpin the phenotypic evolution (13). Combining multiple approaches will lead to cross information, allowing a dissection of the genetic mechanisms of resistance to infections, and contribute to the identification of potential targets of selection for improved resistance (14).

The objective of this study was the identification of the genetic determinants of resistance to vibriosis using the species *C. semilaevis* as a model. With this objective, we sequenced, analyzed and compared the genomes and transcriptomes of selected resistant and sensitive fish. Both the genomic and transcriptomic divergence highlighted the functional potentials of CS/DS metabolism and ECM-receptor interaction in the vibriosis resistance. Additionally, we characterized the expression and defensive functions of crucial genes in the host defense against the bacterial pathogens. These results demonstrated that the selection pressure has acted on specific genes and pathways in mediating the bacterial adhesion and invasion, which may largely account for the improved vibriosis resistance.

## Results and discussion

### Genome-wide selective sweeps and genes relevant to vibriosis resistance

The selection pressure finally acts on phenotype. To accurately detect the genomic signatures of the selection associated with vibriosis resistance, we measured the genome-wide variations between 74 pre-selection and 108 post-selection *C. semilaevis*. From the genome resequencing data, we detected 3,768,965 single nucleotide polymorphisms (SNPs), among which 1,600,893 SNPs were located in the genic regions, including 51,901 nonsynonymous, 131,463 synonymous and 1,417,529 intronic SNPs. In addition, 2,142,956, 9,050 and 15,254 SNPs were located in intergenic, upstream and downstream and unknown regions, respectively (**Supplementary Table S1**).

**Figure 1:**
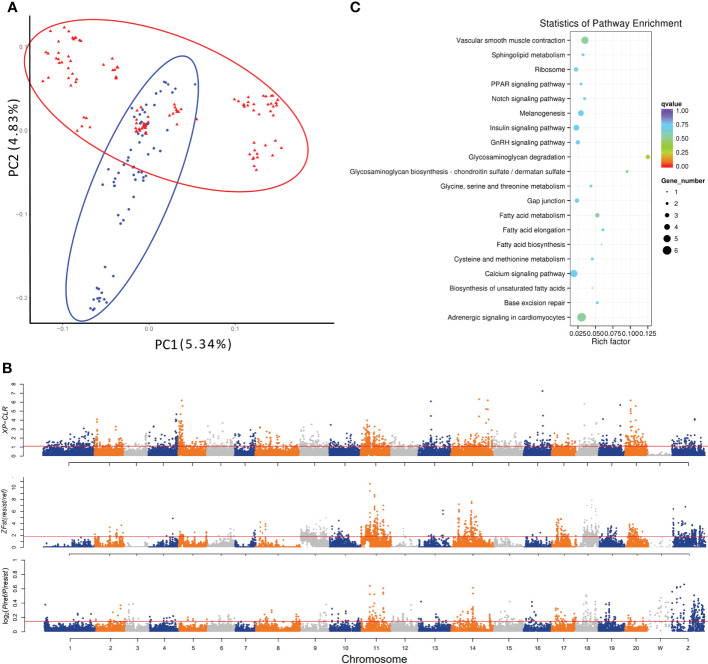
Genetic divergence and genome-wide identification of selective sweeps for vibriosis resistance in *C. semilaevis*. A total of 3,768,965 SNPs (MAF > 5%, missing rate < 10%) identified from 182 samples were used. **(A)** PCA shows a genetic divergence between pre-selected (*ref*) and post-selected (*resist*) individuals. The first and second dimensional coordinates are plotted. Pre-selected and post-selected individuals are shown in blue and red colors, respectively. **(B)** Distributions of XP-CLR, *Z* (*F*_ST_) and *θπ* (in log_2_ (*θπ* ratio (*θπ_ref_*/*θπ_resist_*)) values calculated using a 40 kb sliding window with a step size of 20 kb. The dashed horizontal lines correspond to the top 5% values of each measurement (where XP-CLR was 1.10, *Z*(*F*_ST_) was 1.82, and log_2_ (*θπ* ratio) was 0.1455). **(C)** Top 20 enriched KEGG pathways for the 207 genes under selective sweep, which were simultaneously identified by XP-CLR, *Z* (*F*_ST_) and log_2_ [*θπ* ratio (*θπ_ref/_θπ_resist_*)].

The result of PCA indicated that the pre-selection and post-selection individuals were separately clustered (**Figure 1A**), which was in line with the phylogenetic relationship revealed by the Neighbor-Joining (NJ) tree (**Supplementary Figure S1A**). Some individuals in the two groups were overlap clustered. A possible reason is that all the fish were originated from a relatively small ancestral breeding population, thus some individuals might have a close genetic relationship. This may also partially explain why in the PCA result, the PC2 mainly discriminates the pre- and post-selection individuals. In addition, the genetic stratification was further confirmed using STRUCTURE program, which identified the optimal number of the genetic clusters when the K was set to 2 (**Supplementary Figure S1B**). These results indicated a genetic divergence correlating with the selection to vibriosis resistance in *C. semilaevis*.

The selected genomic regions are expected to have a reduced allele frequency, elevated differentiation, and lower genetic diversity between genetically diverged groups. To detect the genomic regions and genes with selection signatures, we screened the genome using three distinct metrics of selective sweeps, including XP-CLR, *F*_ST_ and nucleotide diversity. First, the XP-CLR approach identified a total of 39.5 Mb genomic regions with selective sweep signals, harboring 2011 gene (XP-CLR value greater than 1.1 (top 5%)) (**Figure 1B**). These genes were enriched in 7 KEGG pathways, including “melanogenesis”, “calcium signaling pathway”, “tight junction”, “phagosome”, “GAG degradation”, “vascular smooth muscle contraction” and “gap junction” (*p* < 0.05) (**Supplementary Table S2**). In addition, calculation of z transformation of *F*_ST_ (top 5%, empirical *F*_ST_ ≥ 1.82) identified 170 selective sweeps in a total length of 45.12 Mb **(** **Figure 1B** **)**. In these regions, we retrieved 2057 genes that were annotated with KEGG pathways such as “lysine degradation”, “notch signaling pathway” and “lysosome” (*p* < 0.05) (**Supplementary Table S2**). Furthermore, we constructed a genome-wide empirical distribution of the log_2_ (*θ*π ratio (*θ*π_ref/_ *θ*π _resist_)) between the pre-selection (ref) and post-selection (resist) groups, and identified 288 selective sweeps (52.98 Mb) that had reduced nucleotide diversity in the post-selection group (5% right tail, where log_2_(*θ*_π_ ratio) was 0.145) (**Figure 1B**). These regions harbored 2302 genes that were overrepresented in various metabolic and signaling pathways such as “phosphatidylinositol signaling system” and “RIG-I-like receptor signaling pathway” (*p* < 0.05) (**Supplementary Table S2**).

We found that most of the selective sweeps were distinctly identified or slightly overlapped, and a total of 5.24 Mb genome sequences were simultaneously identified by the three metrics. A total of 207 genes were in these shared selective sweeps (**Supplementary Table S3**), which were most overrepresented in “GAG degradation” and “GAG biosynthesis-chondroitin sulfate (CS)/dermatan sulfate (DS)” (*p* < 0.05) (**Table 1**; **Figure 1C**). CS/DS are representative sulfated GAGs that are widespread on cell surfaces and are abundant in the ECM, where they have essential functions in tissue development and homeostasis and are among the first host macromolecules encountered by infectious agents (15). These results indicated that mutations affecting genes in the CS/DS metabolism pathways may underlie the changes in the vibriosis resistance and provided clues for the functional characterization of the genes responsible for this trait.

**Table 1:** Enriched KEGG pathways for the genes in selective sweeps simultaneously identified by XP-CLR, *F*_ST_ and nucleotide diversity measurements.

#Term	ID	p-Value	qValue
Glycosaminoglycan degradation	dre00531	0.004092	0.286472
Glycosaminoglycan biosynthesis - chondroitin sulfate/dermatan sulfate	dre00532	0.031101	0.534791
Vascular smooth muscle contraction	dre04270	0.033716	0.534791
Fatty acid metabolism	dre01212	0.035902	0.534791
Adrenergic signaling in cardiomyocytes	dre04261	0.038199	0.534791

### Transcriptional differences between the resistant and susceptible groups

Variation in gene expression patterns often plays a key role in the evolution of many complex phenotypes. To explore whether the gene expressions, especially those in the CS/DS metabolism, were regulated, we performed RNA-seq comparisons in gill and skin between the resistant and susceptible families. Both gill and skin are the surface tissues that directly encounter outside stimulations and act as the first line of defense against pathogens.

**Figure 2:**
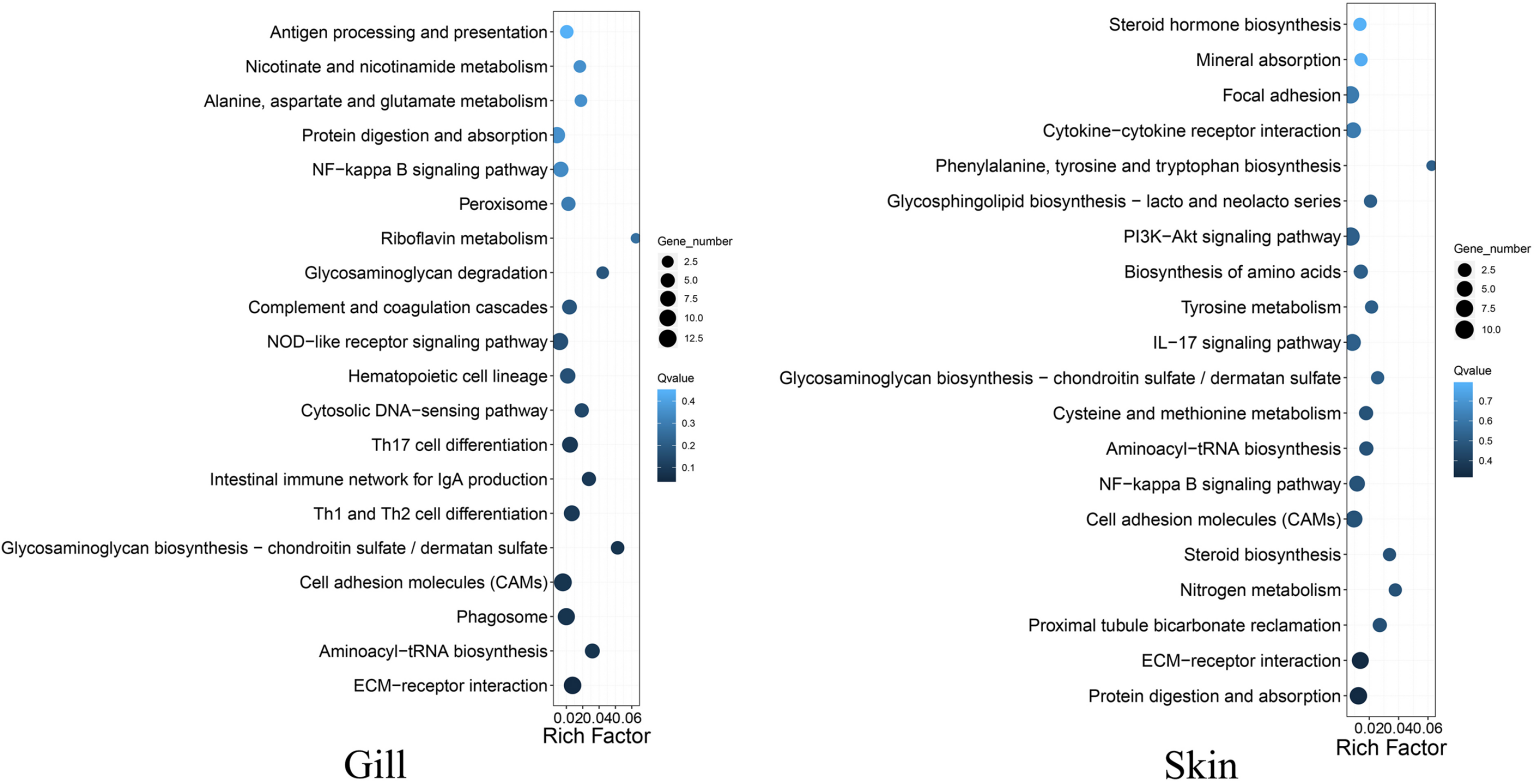
Top 20 KEGG pathways enrichments for the DEGs between the resistant and susceptible families of *C. semilaevis*.

A total of 653 and 1421 differentially expressed genes (DEGs) were identified in gill and skin, respectively (**Supplementary Figure S2**). The DEGs included 367 and 1001 down-expressed, and 286 and 420 up-expressed in gill and skin of the resistant family, respectively. The discovery of more than 1000 transcriptional divergent genes indicated that the resistance against vibriosis in *C. semilaevis* might be controlled by multiple genes. This is in line with the results in fish and mammals that a few genes with large range of immune responses control host defense against foreign organisms (16). Moreover, KEGG analyses allowed an identification of the DEGs significantly enriched for “ECM-receptor interaction” in gill, and in “complement and coagulation cascades”, “cardiac muscle contraction”, “starch and sucrose metabolism” and “aminoacyl-tRNA biosynthesis” in skin (adjusted *p* < 0.05) (**Figure 2**; **Supplementary Table S4**).

Interestingly, we observed that the up- and down-expressed genes in gill were enriched for “GAG biosynthesis-CS/DS” and “GAG degradation”, respectively (**Figure 2**; **Supplementary Table S5**), indicating that the metabolism of CS/DS might be under distinguished regulations between the resistant and susceptible families. These transcriptomic results are as would be predicted from results of the selective sweep analyses, indicating that the artificial selection has substantially changed the genes and gene expressions in the CS/DS metabolism, which may contribute to drive the vibriosis resistance evolution.

CS/DS is a major component of the ECM, which is mainly composed of water, proteins, and polysaccharides. It is notable that the DEGs in both gill and skin were enriched in “ECM-receptor interaction” **(** **Figure 2**; **Supplementary Table S5, S6**), which participates in a wide variety of cellular functions including the homeostasis, inflammation, and response to bacterial infection (17). Our results indicated that this pathway and the involved DEGs might link tightly to the improvement of vibriosis resistance.

### CS/DS metabolism and chst12 gene in vibriosis resistance

Both the selective sweep and transcriptomic analyses pinpointed a conspicuous connection of the biosynthesis and degradation of CS/DS to the evolution of vibriosis resistance (**Figure 1C**, **Figure 2**). CS/DS has a number of useful biological properties for tissue integration including anti-inflammatory activity, water and nutrient absorption, improved wound healing and biological activity that may help to restore arthritic joint function (18). Previous studies have demonstrated that several pathogens including parasites, bacteria, and viruses can utilize cell surface CS/DS chains to attach to and infect host cells (15). For example, CS chains rich in E units can serve as a cell surface receptor in the case of herpes simplex virus (HSV) infection (19). In addition, it has been reported that CS can activate the NF-κB transcription factor in antigen presenting cells and this pro-inflammatory immune response of CS was largely dependent on its molecular size and the degree of acetylation (20).

**Figure 3:**
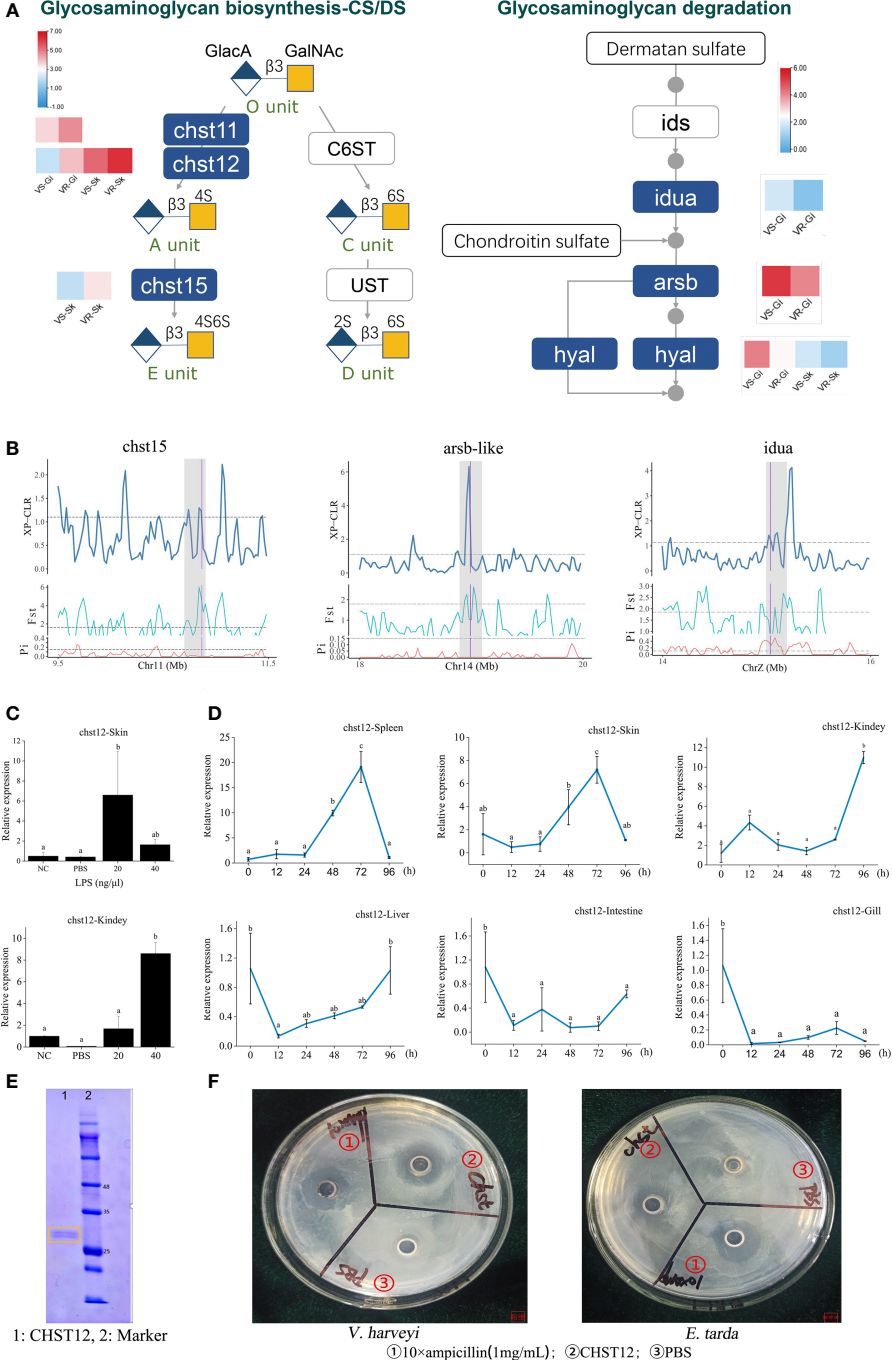
Identification of glycosaminoglycan CS/DS metabolism that contribute to improvement of vibriosis resistance. **(A)** Schematic diagram of pathways for biosynthesis (right panel) and degradation (left panel) of CS/DS chains. Heatmaps show the significantly different expression levels of the genes in the resistant (VR) and susceptible (VS) families. Gi: gill, Sk: skin. Ids: iduronate 2-sulfatase; Ust: uronyl 2-O-sulfotransferase; C6ST: chondroitin 6-O-sulfotransferase-1. O unit: GlacA-GalNAc, A unit: GlacA-GalNAc(4S), C unit: GlacA-GalNAc(6S), D unit: GlacA(2S)-GalNAc(6S), E unit: GlacA-GalNAc(4S, 6S). 2S, 4S, and 6S represent the 2-O-, 4-O-, and 6-O-sulfate group, respectively. GalNAc: *N*-acetylgalactosamine, GlcA: glucuronic acid. **(B)** *chst15*, *arsb-like* and *iuda* genes in glysosaminoglycan metabolism pathways were embedded in selective sweeps. The XP-CLR, *F*_ST_ and *θπ* ratio values are plotted. Genomic regions located above the dashed line (corresponding to the top 5% values) were termed as strong selective sweeps for the post-selection individuals (grey regions). The boundaries of genes are marked in purple. **(C)** Relative expression levels of *chst12* gene after LPS stimulation, with respect to its background expression levels in kidney and skin cells, respectively. Cells were treated with LPS at 28°C for 2h. **(D)** Time-course relative expressions of *chst12* gene in skin, gill, spleen, liver, kidney and intestine after *V. harveyi* infection. Data are means ± S.D., representing average values of three replicates. Different words indicate significant differences (*p* < 0.05). **(E)** Analysis of recombinant Chst12 by 12% SDS-PAGE. **(F)** Antimicrobial activity of recombinant Chst12 against *V. harveyi* and *E. tarda* using Oxford cup method.

The tissue CS/DS content depends on both synthesis and degradation of these molecules. Our RNA-seq data showed that *chst12*, *chst15* and *chst11-like* genes exhibited significantly elevated expressions in the resistant families (**Figure 3A**, left panel). On the contrary, most of the genes pivotal for CS/DS degradation, including alpha-iduronidase (*idua*), arylsulfatase B-like (*arsb-like*) and hyaluronidase-5-like (*hyal5*) were lower-expressed (**Figure 3A**, right panel). Therefore, not only increased biosynthesis, but also decreased their degradation may contribute to increase the CS/DS content in the resistant family. At genetic level, three genes including *chst15*, *arsb* and *idua*, which have undergone selective sweep, are critically important for the CS metabolism (**Figure 3B** **)**. For example, *arsb* is required for the hydrolysis of 4-sulfates of the *N*-acetyl-d-galactosamine-4-sulfate units of CS and DS. Therefore, the genetic changes on these genes may have facilitated the regulation in gene expressions and the evolution of the resistance to vibriosis.

The carbohydrate sulfotransferase (Chst) are key enzymes that can catalyze the transfer of sulfate to position 4 of the N-acetylgalactosamine (GalNAc) residue of CS/DS and play a key role in tissue remodeling (21). The Chst12 is one of the CS structure modifying sulfotransferases, which can effectively regulate the levels of CS synthesis (5). Previous studies showed that inhibition of CHST12 promoted inflammation in human bone diseases (22). In zebrafish, Chst12 and other CS/DS modification enzymes are differentially expressed while CS/DS structure varies significantly during development (23). However, very few studies have investigated the role of the sulfotransferase in the host defense against pathogens. Here we first explored the expression characteristics of *chst12* upon bacterial stimulation *in vitro* and *in vivo*. Results showed that the expression of *chst12* was robustly stimulated by lipopolysaccharide (LPS) in both the skin and kidney cells (*p* < 0.05), whereas its response to PBS was modest (**Figure 3C**). Using the tissue samples removed from the *V. harveyi* infected fish, we observed that the expression of *chst12* varied significantly after the infection. Specifically, in both skin and spleen, the transcript levels of *chst12* gradually increased from 24 hours post infection (hpi), reaching the peak at 72 hpi (*p* < 0.05). In kidney, the peak of expression level appeared at 96 hpi (*p* < 0.05). In gill, intestine and liver, a decreased expression occurred at 12 hpi and maintained at a low level till 96 hpi (**Figure 3D** **)**. These results indicated that the infection may stimulate the expressions of *chst12* in skin, kidney and spleen, while the expressions in gill and intestine were inhibited. The differential expression patterns in different tissues also indicated that *chst12* gene is a highly responsive gene to the infection of *V. harveyi*, and that CHST12 may play roles in both mucosal and systemic immune processes against the bacterial invasion. Further studies need to be performed to illustrate the specific function of *chst12* gene in the immune responses in different tissues.

We further constructed the Chst12 recombinant protein using *Pichia pastoris* KM71. The molecular weight of the recombinant Chst12 was about 28-30 kDa, which was verified by 12% SDS-PAGE (**Figure 3E**). Using the Oxford cup method, we found that recombinant Chst12 had an obvious inhibitory ability against both *V. harveyi* and *Edwardsiella tarda* (**Figure 3F**). Thus, *chst12* gene might play dual roles in the vibriosis resistance both indirectly, by regulating the CS/DS biosynthesis and directly, by inhibiting the bacterial growth. To the best of our knowledge, this is the first report that the *chst12* gene has significant bactericidal impact upon infectious bacterial pathogens. Previous studies have shown that the Chst proteins may play important regulatory roles in a variety of human disease and cancers (24). In addition, evidence showed that Chst genes had the antiviral function and enhanced resistance to white spot syndrome virus in *Procambarus clarkii* (25). Taken together, we identified Chst12 as a significant CHST member which plays an anti-infection role in vibriosis resistance. These results demonstrated that the artificial selection for vibriosis resistance has likely acted at least partly on the genes for CS/DS metabolism, in which the defenses preventing the establishment and invasion of pathogens are caused mainly by fine-tuned modulation of CS/DS and gene antibacterial activity.

### ECM-receptor interaction in vibriosis resistance

**Figure 4:**
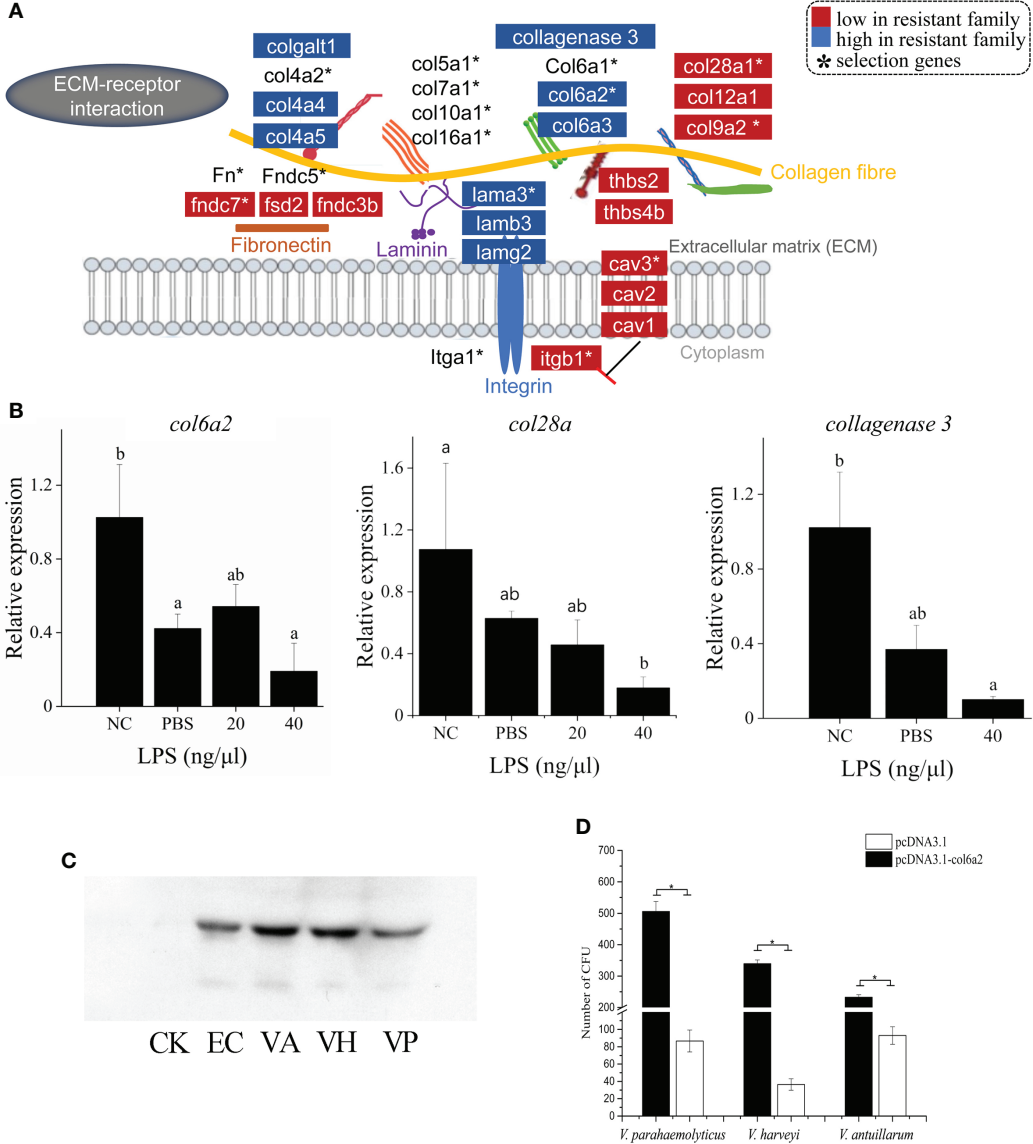
ECM-receptor interaction has strong associations with vibriosis resistance and identification of Col6a2 as a *Vibrio* receptor for *C. semilaevis*. **(A)** Schematic of the genetic and transcriptomic divergence in ECM-receptor interaction. Red and blue colors indicate the lower and higher expressed genes in the resistant family compared to susceptible family (*p* < 0.05). * indicates the genes under selective sweeps. **(B)** Relative expressions of *col6a2*, *col28a* and collagenase 3 gene in skin and kidney cells after LPS stimulation. Cells were treated with 20 and 40 ng/μL LPS at 28°C for 2h. The alphabets a, b and c indicate significantly different expressions among the samples (P < 0.05). **(C)** Western Blot analysis showed that Col6a2 binds to three *Vibrio* pathogens, including *V. anguillarum,* (*VA*); *V. harveyi,* (*VH*); *V. parahaemolyticus,* (*VP*); *E. coli* (EC) and control (CK). **(D)** Transfections of HEK293T cells with *col6a2* enhanced the *Vibrio* adhesions. * indicate significant differences (*p* < 0.05) of the transfected versus untransfected cells using one-way ANOVA.

The genetic and transcriptomic analyses also presented an emphasis on the functional potential of “ECM-receptor interaction” in the vibriosis resistance (**Figure 2**), involving 15 ECM genes under the selective sweeps, and 16 and 20 DEGs in gill and skin, respectively (**Figure 4A**; **Supplementary Table S7**). The intersection of DEGs and selection genes consisted of seven genes, including *col6a2*, *col9a2*, *col28a*, *lamb3*, *fndc7*, *cav3* and *itgb1* (**Figure 4A**).

ECM composing of several protein components, such as collagen (Col), laminin (Ln), fibronectin, is a complex and dynamic structure that provides the scaffold and surface where complex interactions between invading pathogens, host tissues and immune cells occur (26). A study in oyster has reported that the responses to *Vibrio tasmaniensis* LGP32 infection was characterized by genes in ECM remodeling and other four categories (27). Accumulating evidence have shown that bacterial pathogens bind to different ECM proteins and adhesive matrix molecules, to effectively establish tissue adherence and invasion (28). In this context, Lnα2 acts as a bridge between the host cell and the pathogens, including group B *Streptococcus*, and *Staphylococcus aureus* (29). Similarly, fibronectin has been reported to have a bridging function in the invasion of *S. aureus* (30).

We found that different types of ECM genes systematically exhibited different expression patterns. For example, the Ln protein family comprising about 20 glycoproteins, assemble into a cross-linked web and interweave with the type IV collagen network in basement membranes (31). The Ln-5 (α3β3γ2) isoform is the dominant form that are distributed in the skin in vertebrates. We observed that all the lnα3, β3 and γ2 genes showed higher expressions in the resistant family (**Figure 4A**), which may indicate an enhanced ECM structure as a physical barrier against the vibrio invasion.

Collagens, constituting of different types that can be subdivided into fibril-forming collagens, network-forming collagens, fibril-associated collagen with interrupted triple helices (FACIT), transmembrane collagens and finally multiplexins. A recent study for steelhead trout (*Oncorhynchus mykiss*) reported that the resistant fish have a different response to parasite infection at the tissue level with the collagenous stratum compactum acting as a barrier preventing parasite spreading (32). We found that the network-forming collagens (type IV collagen) *col4a4* and *col4a5*, which are the major nonfibril structural component of basement membranes, were up-expressed in skin tissue of the resistant families; The fibril-associated collagens with interruptions in their triple helices (FACITs), such as *col6a2*, *col6a3, col12a1* and *col28a1* showed down-expressions in skin tissue; All the fibril-forming collagens (e.g., types I, II, III collagen) levels were not different (**Figure 4A**).

In addition, several other collagen related genes were also differentially expressed. For instance, procollagen galactosyltransferase 1-like, which is involved in the biosynthesis of collagen type IV and facilitates the formation of collagen triple helix, was up-expressed (**Figure 4A**). The collagenase 3-like gene, which encodes an enzyme that degrades a variety of ECM proteins, including fibronectin, laminin and types III, IV, IX, and X collagen, was also up-regulated (**Figure 4A**). Therefore, the fine-tuned expressions of these ECM genes indicated ECM remodeling and an alteration in the ECM architecture, which may lead to an enhancement of the barrier function. Furthermore, we measured the expression patterns of three ECM genes including *col6a2*, *col28a* and collagenase3, in response to LPS stimulation in skin cells. All these genes exhibited significant decreased levels with LPS with a higher concentration (40 μg/μL) (*p* < 0.05) (**Figure 4B**), suggesting that they are responsive to bacterial simulations.

The first event in bacterial invasion requires attachment of the bacteria to the host cells. Pathogens usually take advantage of existing receptor proteins to facilitate opportunistic penetration in hosts. To identify the candidate receptor protein for *Vibrio* species, we exploited the role of an ECM gene *col6a2*, which was down-expressed in both gill and skin of the resistant family, in mediating the bacterial adhesion to host cell. First, to assess whether bacteria can bind to Col6a2, we mixed the recombinant Col6a2 protein with different *Vibrio* pathogens, including *V. parahaemolyticus*, *V. anguillarum* and *V. harveyi*. After removing the unbound Col6a2 protein, the bindings were measured using Western Blot analysis. Clear target bands were detected in all cases (**Figure 4C**), indicating that these Vibrio pathogens could directly bind to Col6a2 protein. In addition, bacteria-cell adhesion assays based on transfected *col6a2* in HEK293T cells showed that the number of adherent bacteria were significantly increased after the transfection, suggesting that the overexpression of Col6a2 could significantly improve the adhesions of all the three *Vibrio* species to HEK293T cells (**Figure 4D**). These results showed the Col6a2 had extracellular adhesion activity to *V. parahaemolyticus*, *V. anguillarum* and *V. harveyi*, thus may act as a *Vibrio* acceptor that can enhance the bacterial-cell adhesions. Taken together, our data validated that Col6a2 may play a bridging role between the *Vibrio* pathogens and the host cell, and the differential expression patterns of the *col6a2* between the resistant and susceptible families may partly account for their different resistance to *Vibrio* infection.

Together with the multiple levels of evidence and previous study linking ECM genes as a preferred target for Gram-negative bacterial adhesion (29, 30), our data suggest that modulation of ECM structure might be an important tissue protective mechanism contributing to vibriosis resistance. We identified Col6a2 as a receptor for *Vibrio* pathogens, and its lower abundance may limit the *Vibrio* adhesion and invasion in the resistant family.

## Conclusions

In this study, we presented the genomic selective signatures and transcriptomic divergence underlying the vibriosis resistance for the *C. semilaevis*. Our results revealed that the selection pressure for resisting *Vibrio* infection may preferentially target genes in the CS/DS metabolism and ECM-receptor interaction pathways, both of which work in mediating bacterial adhesions and invasions and act as the first barrier of the host defense system. Furthermore, we characterized *chst12* and *col6a2* as critical genes with important functional implications for defense against bacterial infections. These results demonstrated that *C. semilaevi* evolved tissue protective mechanisms as a first defense line preventing invasive vibrio diseases. Our findings provide critical genetic resources facilitating breeding, as well as important knowledge to improve the prevention and treatment of fish infectious diseases.

## Materials and methods

### Selective breeding and sample preparation

The selective breeding of the vibriosis resistant and susceptible families for *C. semilaevis* were performed as previously described (12). Briefly, we first identified the genetic sex of parental fish by a sex-specific AFLP marker (33), and constructed full-sib families by strip spawning. Each family was tagged with visible implant elastomers and reared in several common tanks under a flow-through system. The pedigree information of each family was precisely recoded to trace their lineages. When fish reached at average size of 10-12 cm, challenge tests were performed by intraperitoneal injection with a medial lethal dose (LD_50_) of *V. harveyi* ATCC 33843 (12, 34). We recorded the mortality of each family, and the families with a survival rate > 80% and < 30% were considered as Vibrio resistant (VR) and susceptible (VS) families, respectively.

The artificial selections have been performed for successive five generations, and the generated VR and VS families were used for transcriptomic sequencing and comparison. We also sampled the fish in the challenge experiment to analyze their time-course immune responses after *V. harveyi* infection. In addition, to identify the genomic divergence and signatures of selective sweeps underlying the resistance variation, we conducted genome resequencing for 182 tongue soles, including 74 tongue soles from the pre-selection breeding population and 108 fish from the post-selection resistant families, which were sampled in 2012 and 2018, respectively.

### Genome re-sequencing and genotyping

Genomic DNA was extracted from the fins using DNeasy Blood & Tissue Kit (Qiagen, Germany). PE libraries with an insert distance of 300 bp were constructed according to the standard protocol (Illumina, USA). The sequencing was performed on Illumina HiSeq platform, producing raw reads in 2×150 bp. The low quality reads were detected and filtered using QC-Chain (35). Finally, the resequencing of the 182 fish yielded a total of 1.39 Tb high-quality data with an average sequencing depth of 13.8 × (**Supplementary Table S8**).

We used the BWA software (36) to align the clean reads to the reference genome (NCBI Accession No. GCA_000523025.1), with an average mapping rate of 97.79% (**Supplementary Table S8**). The variants calling was performed with SAMtools (37) and GATK (38) with default parameters, respectively. SNPs identified by both the methods were retained for further analysis. Then the SNPs with minor allele frequency (MAF) > 0.05 and missing rate < 10%, and no departure from Hardy-Weinberg equilibrium (*p* < 0.001) were used for further analyses.

### SNP annotation

We used ANNOVAR (39) to annotate the SNPs as coding regions, UTRs, upstream or downstream regions (within 1 kb region from the transcription start or stop site), and intergenic regions. Exonic SNPs were further categorized into synonymous (causing no amino acid changes), nonsynonymous (causing amino acid changes), stop gain or stop loss ones. The SNPs-related genes were functional annotated by KEGG database using BLAST.

### Population structure analysis

Principal-component analysis (PCA) of the genetic divergence between the pre-selection and post-selection individuals was performed using GCTA (40) and the first two dimensional coordinates were plotted. An individual-based neighbor-joining tree was constructed using TreeBest (v1.9.2) (41) according to a *p*-distance matrix with a bootstrap value of 1,000. The genetic structure was also examined using the software STRUCTURE (38), setting the pre-defined genetic clusters (K) from 2 to 5. We ran the analysis with 10,000 iterations.

### Genome-wide scan of selective sweeps

To detect the candidate selected regions between the pre-selection (original, ORI) and post-selection (resistant, RES) fish, we firstly used a cross-population composite likelihood approach XP-CLR (Chen et al., 2010) to compare the allele frequency distributions with parameters of ”-w1 0.005 100 100 –p0 CHR# 0.8”. Then we used the program VCFtools (v0.1.14) (42) to estimate the fixation index (*F*_ST_) and the nucleotide diversity (*θπ*), which was represented by the log_2_(*θπ* ratio) of *θπ_ORI_/θπ_RES_*, throughout the whole genome. A 40 kb non-overlapping window with a step size of 20 kb, was used to screen the whole genome and the windows containing more than 10 SNPs were retained. Adjacent windows were merged into a single selective sweep if their distance was less than 200 kb. The windows with top 5% of the maximum XP-CLR, *F*_ST_ and log_2_(*θπ* ratio) values were deemed as candidate significant selective sweeps and genes in these regions were defined as selection genes. Additionally, the selection genes locating in the overlapping selective sweeps identified by the three metrics were subjected to KEGG and GO enrichment analyses.

### RNA-seq and comparative transcriptomic analyses

To characterize and compare the gene expression patterns, we collected gill and skin tissues from the resistant and susceptible families, respectively. Three replicates for each tissue samples were used for total RNA extraction with TRIzol (Invitrogen, USA). Pair-ended (PE) RNA-seq libraries were constructed using the Truseq mRNA stranded RNA-Seq Library Prep Kit (Illumina, USA) according to the standard protocol. Sequencing of the 30 libraries was conducted with a BGI-Seq500 sequencing platform, generating raw reads with a read length of 2 × 100 bp and an insert size of 350 bp. The quality control of the raw data was performed with RNA-QC-Chain (43) to remove the ambiguous N’s, adaptor reads, low quality reads with more than 20% of the bases having a quality score < 20. Finally, we obtained 62.26-79.69 million raw reads per sample, amounting to a total of 86.47 Gb clean data **(** **Supplementary Table S9**). The raw reads were deposited at the NCBI sequence read archive (SRA) under project number PRJNA785712.

We aligned the clean reads to the reference genome of *C. semilaevis* (NCBI Accession No. GCA_000523025.1) using BWA (36). The mapping rates varied from 83.9% to 93.1%, averaging 88.6% (**Supplementary Table S9**). Fragments per kilobase per million mapped sequence reads (FPKMs) value for each gene was calculated with RSEM (v1.2.12) (44). Then we used NOIseq (45) to detect the DEGs, which were defined following the criteria of |log_2_(Fold Change)| ≥ 1, with a probability ≥ 0.9. Hierarchical heatmaps of the gene expression levels were constructed with Euclidean distance using the Cluster (v3.0) (46).

### KEGG enrichment analyses

We conducted KEGG and GO enrichment analyses using phyper in R software, with *Danio rerio* (dre) as the reference species for the KEGG analyses. KEGG pathways and GO terms with *p-*values less than 0.05 were considered enriched, and with FDR of the *p*-value (*q*-value) less than 0.05 to be significantly enriched.

### Cell culture and LPS treatment

The skin and kidney cells were cultured using similar methods as previously described (47). Briefly, the cells were derived from the corresponding tissues of the tongue sole, and were maintained at 24°C in L-15 medium with 20% fetal bovine serum (FBS), 100 IU/mL penicillin and 100 μg/mL streptomycin. Cells were subcultured over 3-4 days using standard procedures, and then plated on 12-well culture plates at a density of 3×10^5^/well to form a complete monolayer (34). After 24 h, LPS (Sigma-Aldrich, USA) was added to reach final concentrations of 20 and 40 ng/μL, respectively. The control group was treated with PBS. The cells were sampled for RNA isolation at 24 h post treatment.

### *V. harveyi* challenge experiment

To investigate the time-course transcriptomic responses to *V. harveyi* infection *in vivo*, we performed a *V. harveyi* challenge test as previously described (34). Briefly, Around 50 fish were intraperitoneal injected with 1.0 × 10^4^ CFU of a 24 h bacterial culture. Another 50 fish were injected with PBS as the control group. Five individuals were sampled at 0, 12, 24, 48, 72 and 96 hours post infection (hpi). Skin and gill tissues were removed and used for RNA extraction and qPCR analyses.

### Quantitative real-time PCR

Total RNAs were extracted using Trizol and reverse transcribed into cDNA with the PrimeScript™ RT reagent Kit with gDNA Eraser (Takara, Japan). The gene expression levels were measured with quantitative real-time PCR (qPCR) using the 7500 Real-Time PCR System (Applied Biosystems, USA). The reaction system consisted of 1 × SYBR Premix Ex Taq, 200 nM each primer, 1 × ROX Reference Dye II (Takara, Japan) and 1 µL of the cDNA template in a final volume of 20 μL, with three replicates for each sample. The PCR conditions were performed as 95 °C for 30 s, followed by 40 cycles for 5 s at 95 °C, and 60 °C for 33 s. The relative expression was analyzed with the 2^-ΔΔCt^ method and the statistical significance (*p* < 0.05) was determined by one-way analysis of variance (one-way ANOVA), followed by a two-sided Dunnett’s *post hoc* test.

### Recombinant protein expression and purification in *Pichia pastoris*

Based on the information of Chst12 and optimal codons of *Pichia pastoris*, codon optimized *chst12* gene sequence was synthesized and cloned into pMV vector by the Beijing Genomics Institute. The plasmid containing the codon optimized *chst12* sequence was named pMV-chst12. Then, a pair of primer 9k-chst12F/9k-chst12R was synthesized and used to amplify the target sequence. The product was ligated into the linearized vector pPIC9k precut with *EcoRI* and *NotI* to construct the recombinant plasmid. The resulting constructs pPIC9k-Chst12 was transformed into the *E. coli* DH5α and verified by sequencing. The recombinant plasmid pPIC9k-Chst12 was extracted and linearized with *SalI* followed by transformation with *Pichia pastoris* host strain KM71 using PEG1000 method (48).

The cDNA encoding CDS without signal peptide of Col6a2 was amplified and cloned into T1 vector, and then transformed into *E. coli* DH5α. The *col6a2* with HIS tag was inserted into pic9K Vector (*EcoRI* site) with the help of ClonExpress^®^ Ultra One Step Cloning Kit (Vazyme, China). The positive clones were confirmed using sequencing, the recombinant expression plasmid was extracted and transformed into GS115 using Quick & Easy Yeast Transformation Mix (Takara, Japan).

Transformants were selected for their ability to grow on histidine-deficient minimal dextrose agar plates. In addition, isolation of genomic DNA was performed, and PCR amplifications were then carried out to select positive clones according to Invitrogen’s recommendations with a pair of primers (5’AOX1/3’AOX1). For each positive clone, small-scale expression trials were initially performed to identify the most productive transformants and secretion of Chst12 was determined by SDS-PAGE using 10% (w/v) separating gel and 5% (w/v) stacking gel at 96 h after induction with methanol. After treatment with methanol at the final concentration of 1% for 4 days, the cells were pelleted out from the culture medium by centrifugation at 8,000 r/min for 10 min at 4 °C. The supernatant was used to purify the recombinant Chst12 by affinity chromatography using Ni-NTA-agarose resin (49). The purified protein was identified by 12% SDS-PAGE. Primer sequences were listed in **Supplementary Table S10**.

### Antibacterial assay

The antibacterial activity of recombinant protein was tested by the Oxford-cup method. The *V. harveyi* ATCC 33843 and *E. tarda* H1 were cultured in LB medium to OD600 nm = 0.5, and then take 100 μL to spread LB plate. Then placed the sterilized Oxford cups vertically on the surface of the plates. 100 μL 1 mg/mL Ampicillin, 50 μg/mL recombinant protein and PBS were filled into the cups respectively. The plates were cultivated at 37°C for 12 h and then halo of growth inhibition were observed.

### Bacteria adhesion to recombinant Col6a2 protein

Three pathogenic *Vibrio* species, including *V. parahaemolyticus, V. anguillarum*, and *V. harveyi*, were cultivated overnight. Then 900 μL bacteria were then combined with 100 μL of recombinant Col6a2 protein and incubated at room temperature (25°C) for 40 min. After centrifugated at 2,000 rpm for 1 min, the precipitate was recovered and submitted to Western blot after being centrifuged at 2,000 rpm for 2 min. SDS-PAGE was used to separate the samples, which were then transferred to the nitrocellulose filter membrane (300 mA for 40 min). The membrane was blocked for 2 h with 5% (w/v) nonfat milk, washed three times with TBST (TBS containing 0.05 percent Tween-20), and then incubated overnight at 4°C with mouse anti-His-tag antibody (the primary antibody, diluted 1: 1000), followed by 1 h incubation with goat anti-mouse HRP-conjugated IgG (the secondary antibody, diluted 1: 1000). After that, the protein was stained with DAB (3, 3’-diaminobenzidine) solution for 10 min. The primary and secondary antibodies were purchased from Beyotime Biotechnology (China).

### Bacteria-cell adhesion assays

Human embryonic kidney cell line HEK293T were purchased from Procell (China) and were routinely cultured following American Type Culture Collection (ATCC) culturing conditions, in Dulbecco’s modified Eagle medium (supplemented with 10% Foetal Bovine Serum and 1% penicillin/streptomycin) at 37°C with 5% CO_2_. The plasmids pcDNA3.1-col6a2 (GFP-tagged) was constructed and used for the overexpression of Col6a2. The transfections of recombinant vector (pcDNA3.1-col6a2) were performed using Lipo8000™ transfection reagent (2 μg DNA and 6 μL Lipo8000) (Beyotime Biotechnology, China). After transfection, the cells were treated with 500 μg/mL G418 for 4 weeks to obtain stable cell lines. All treated samples were observed and captured under Nikon ECLIPSE TE 2000-U fluorescence microscope.

The bacterial and cell adhesion assays were then performed. Briefly, 2×10^5^ HEK293T cells per well were seeded into 24-well plates. After culturing overnight, the plates were gently washed with PBS to remove the non-adherent cells. Then 50 μL of the diluted bacteria (10^2^-10^3^ CFU/mL) was mixed into the cell well and incubated for 30 min. After that, the plates were gently washed with PBS to remove the non-adherent bacteria. The attached cells were then gently harvested with Cell Scrapers and transferred to coated plates with LB for *V. parahaemolyticus* RIMD 2210633 and *V. anguillarum* PF4-E2-R4, and with trypticase soy broth for *V. harveyi* ATCC 33843. After being cultured at 28 °C or 37 °C for 12 h, the colonies were counted with Gel-Pro Analyze v4.0 (Media Cybernetics, USA). Statistical significance (*p* < 0.05) was determined by one-way ANOVA.

## Data availability statement

The datasets presented in this study can be found in online repositories. The names of the repository/repositories and accession number(s) can be found below: https://www.ncbi.nlm.nih.gov/, PRJNA542202.

## Ethics statement

The animal study was reviewed and approved by the Animal Care and Use Committee of the Chinese Academy of Fishery Sciences.

## Author contributions

QZ and SC conceived the study and designed the analytical strategy. QZ, LW, and QHZ performed animal work and prepared biological samples. QZ analyzed the data. YC performed the cell culture, recombinant protein expression and cDNA transfection experiment. ZC, XM, and JW performed the qPCR experiments. QZ and SC wrote the manuscript. All authors contributed to the article and approved the submitted version.

## Funding

This work was supported by National Natural Science Foundation of China (grant number 31973006); Central Public-interest Scientific Institution Basal Research Fund, CAFS [grant number 2020TD20] and Taishan Scholar Climbing Project of Shandong Province of China.

## Conflict of interest

The authors declare that the research was conducted in the absence of any commercial or financial relationships that could be construed as a potential conflict of interest.

## Publisher’s note

All claims expressed in this article are solely those of the authors and do not necessarily represent those of their affiliated organizations, or those of the publisher, the editors and the reviewers. Any product that may be evaluated in this article, or claim that may be made by its manufacturer, is not guaranteed or endorsed by the publisher.
