# Supplementary figures and images for: Genomics and transcriptomics reveal new molecular mechanism of vibriosis resistance in fish
Source: Front Immunol. 2022 Sep 29;13:974604. doi: 10.3389/fimmu.2022.974604 (PMC9592550; doi:10.3389/fimmu.2022.974604)

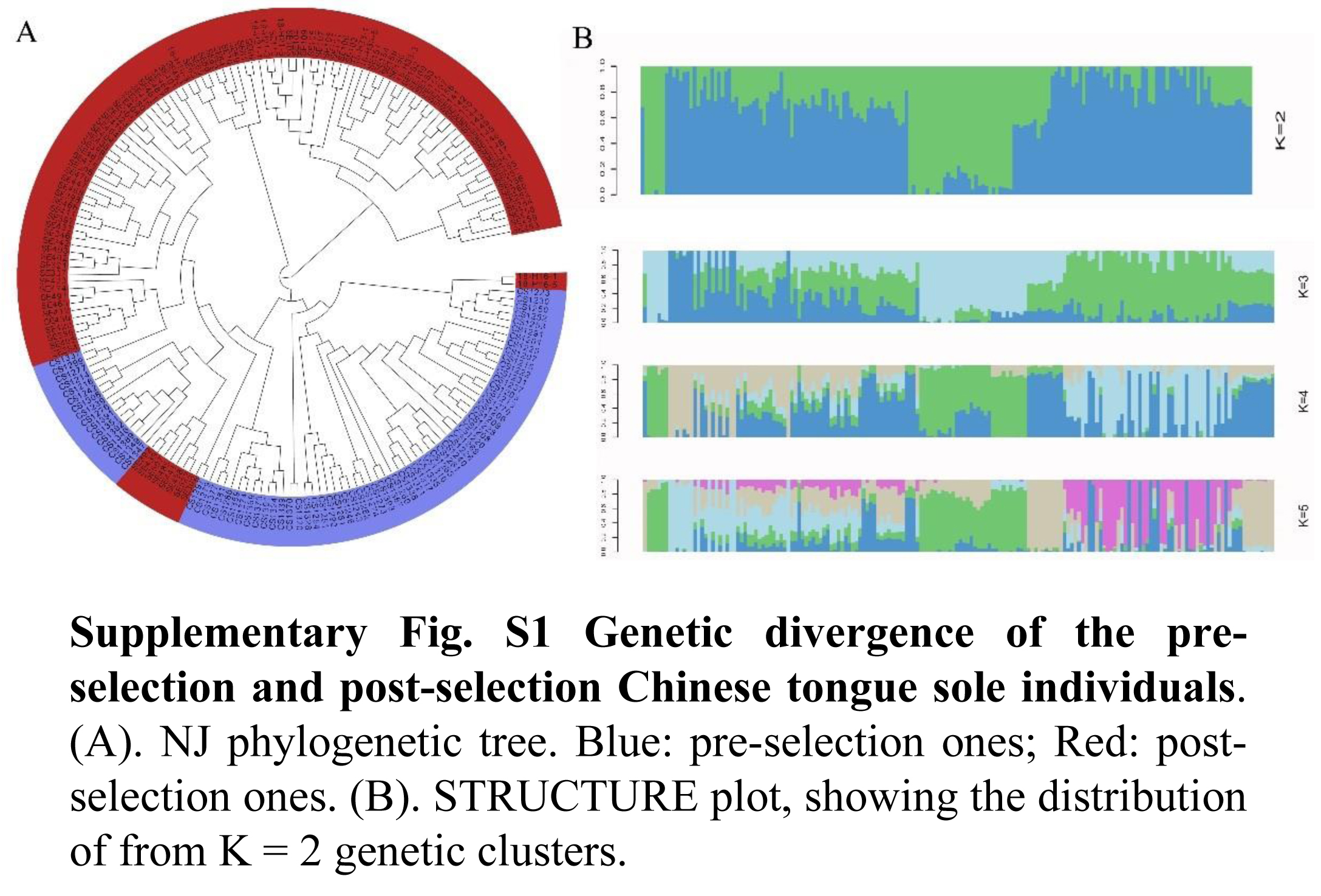

Supplement: Supplementary file 2 [file Image_1.jpg]

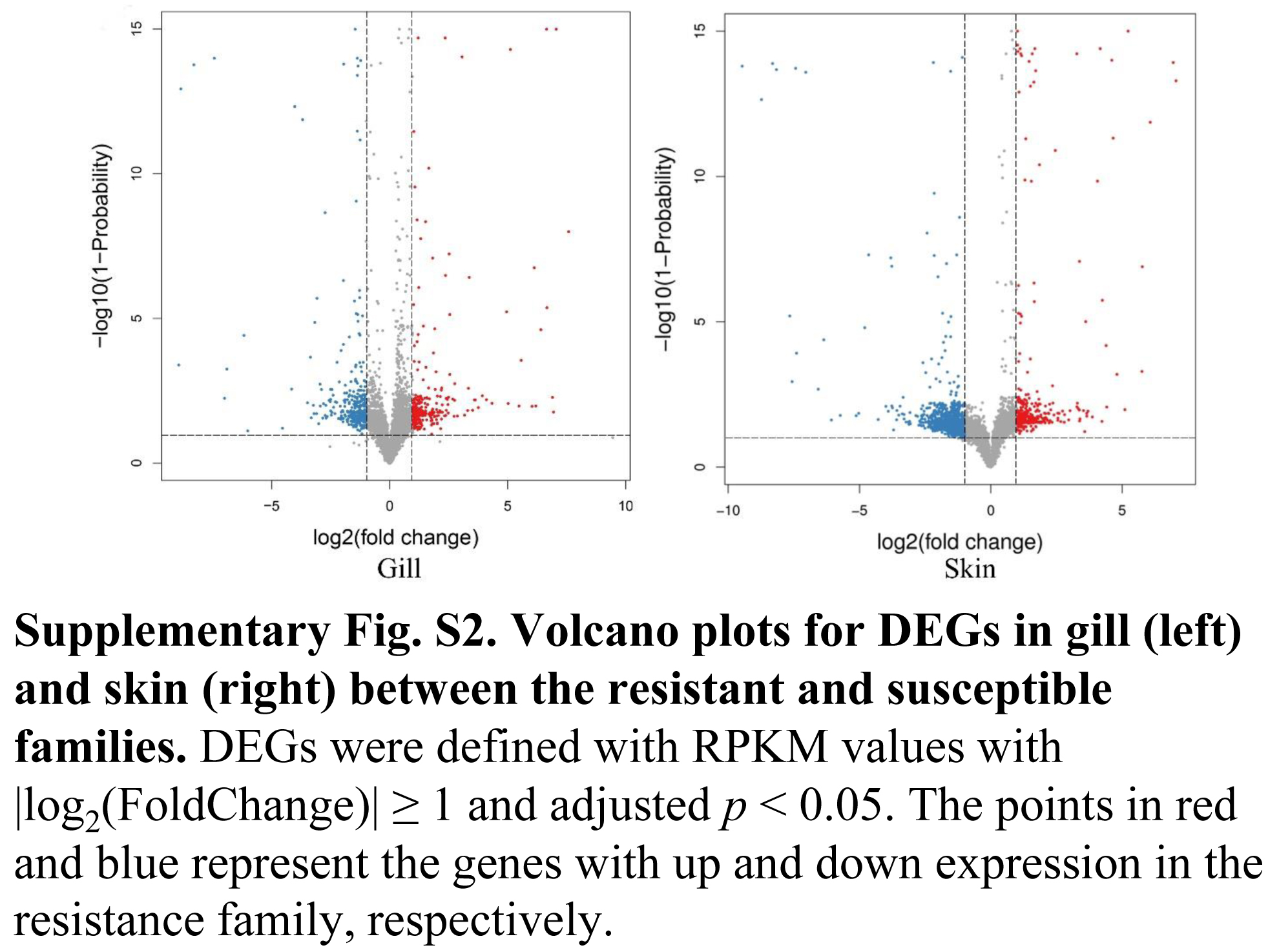

Supplement: Supplementary file 3 [file Image_2.jpg]
